# Effect of a Long Exercise Program in the Reduction of Musculoskeletal Discomfort in Office Workers

**DOI:** 10.3390/ijerph17239042

**Published:** 2020-12-04

**Authors:** Alberto Villanueva, Juan Rabal-Pelay, César Berzosa, Héctor Gutiérrez, Cristina Cimarras-Otal, Belén Lacarcel-Tejero, A. Vanessa Bataller-Cervero

**Affiliations:** 1 Universidad San Jorge, Campus Universitario Villanueva de Gállego, Autov. A-23 Zaragoza - Huesca, Km 299, Villanueva de Gállego, 50830 Zaragoza, Spain; villacos2612@gmail.com (A.V.); jrabal@usj.es (J.R.-P.); cberzosa@usj.es (C.B.); hgutierrez@usj.es (H.G.); ccimarras@usj.es (C.C.-O.); 2 Hospital MAZ, Av. Academia Gral. Militar, 74, 50015 Zaragoza, Spain; blacarcel@maz.es

**Keywords:** muscle tone, workplace, trapezius, shoulder, neck, upper back, lower back

## Abstract

The purpose of this study was to assess the effect of a six week exercise program to reduce the muscle tone of the trapezius and musculoskeletal discomfort (MED) of office workers. Twenty-six workers performed an exercise program based on: (1) stretching of cervical and/or dorsal region; (2) joint mobility of shoulders and rachis; (3) strengthening deep stabilizer and core muscles; and (4) scapula stabilizing exercises. A Myoton device was used to evaluate trapezius tone and the Cornell Musculoskeletal Discomfort Questionnaire was used to assess changes in MED at three points of evaluation: at the beginning (Pre_1) and at the end of the workday (Post_1), and after the training program (Pre_2). The Wilcoxon test and Cohen’s d were performed to examine differences and effect sizes between evaluations. Main results show that trapezius tone remained constant during the workday, but decreased in the dominant upper trapezius (*p* = 0.003, ES = −0.60) and increased in the non-dominant middle trapezius (*p* = 0.016, ES = 0.45) after the exercise program, which eliminated significant muscle asymmetries. MED significantly decreased in the neck (*p* = 0.027, ES = −0.60) and upper back (*p* = 0.046, ES = −0.67). In conclusion, MED appears to improve in office workers after a six week training program, which may be explained by a decrease in trapezius tone and increase in the left middle trapezius tone.

## 1. Introduction

In Europe, musculoskeletal disorders (MSDs) are one of the most expensive work-related diseases for the health care system and companies. Interventions focus on treating the symptoms of these MSDs, which helps to improve the quality of life of workers and may increase their working performance [1,2,3,4,5].

MSDs include pain or other functional diseases of joints, or other damage in connective and/or muscle tissues. Several personal and environmental factors can influence the origination and development of MSDs and, furthermore, these diseases can cause sick leave and require medical treatment [6]. Research is now focused on the causal relationship between an employee’s job and MSDs, the identification of risk factors for MSDs, and the design of specific preventive interventions.

Prolonged sitting positions, such as those experienced by office workers, are a risk factor for MSDs. Research results associate these sitting postures with MSDs in the neck and shoulders of office workers [7]. Different studies have found percentages between 17% and 27% of office workers reporting a new pain in the neck or lumbar region [8,9]. The one-year estimated incidence of neck pain ranges between 10% and 21%. This incidence is higher in office workers than in other occupations.

The muscle tension perceived by a person could be the main predictor of the subsequent onset of an MSD that causes pain in the shoulder, neck, and lower back regions. Muscular tension symptoms are common among office workers and can be described as a feeling of stiffness, tightness, or fatigue depending on the sensory experience of each person [10].

Studies that analyze the relationship between neck pain and scapular disfunction have begun to emerge. The association between posture and abnormal movements of the shoulder girdle with a pathology related to the glenohumeral joint have been well stablished in the literature [11]. Other studies have shown that excessive dorsal kyphosis can cause discomfort in the upper back, which may indirectly influence the development of a shoulder compression syndrome. This could be due to a restriction in the range of movement of the joint as a consequence of the limitation of the lumbar back extension and/or as a cause of scapular dyskinesia [12].

Several programs of exercise have been proposed as possible interventions for the reduction of MSDs in computer users and for the promotion of movement. It can be hypothesized that a short-term intervention (<10 days) of regular exercise during work time could reduce MSDs and improve postural immobility [13,14]. Although there is no clear consensus about the suitable methods of exercise for patients suffering from pain at work, three alternatives were found in the literature: (1) exercise based on scapular stability for the shoulder and neck region; (2) lower trapezius strengthening work; and (3) exercise aimed at increasing the strength of the neck’s superficial and deep musculature. The combination of these three exercises can reduce dorsal kyphosis, increase shoulder stability, and generally improve the functionality and symptomatology of office workers with MSDs in the neck region [15,16]. Numerous studies have been conducted in which exercise programs for the reduction of musculoskeletal discomfort have been developed. However, the validity of these studies is not always clear. Therefore, there is a lack of consolidated evidence for the best intervention programs for the prevention and reduction of the pain in office workers.

A program designed to reduce muscle stiffness and strengthen specific muscle groups, developed at the employees’ own work site, can be effective in reducing pain in the neck, shoulders, and lower back [17]. However, there are a lack of studies that assess the effect of prolonged sitting on the modification of the muscle tone of an employee’s trapezius or if the muscle tone is changed following an exercise program.

The main hypothesis of this study is that the application of a six week exercise program at the workplace will reduce the muscle tone of the trapezius and the musculoskeletal discomfort of office workers.

## 2. Materials and Methods

The present study had a one-group quasi-experimental design. The sample consisted of office workers of a health company in Spain. The development of the study respected ethical considerations established by the World Health Association in the Helsinki Declarations and by the Deontological code of the Spanish General Council of Physiotherapist Colleges. The study was approved by the Ethical Committee of the institution. All participants were recruited by means of an internal company note. They were duly informed about the study procedure and the participation was voluntary.

### 2.1. Participants

All workers that participated in the study were assessed by a doctor with more than 5 years’ experience to determine their suitability for inclusion and to discard pathologies that could interfere, such as cervical hernias, root pains, and shoulder compression syndrome.

The participants had similar working conditions and used an ergonomic adjustable chair with wheels. They used a computer screen and keyboard, and a mouse located to the right of the keyboard. The screen was in front of the desk with the top border at eye level.

The following inclusion criteria were considered: (1) age between 20 and 65 years old at the time of the study; (2) working during most of the work day in a prolonged sitting position; (3) 7 or 8 h working time in the morning shift per day; and (4) more than one year working in the same position.

The exclusion criteria were: (1) scoliosis diagnosed by a physician; (2) previous back surgery; (3) suffering from cervical rachis diseases; (4) treated by subacromial infiltration or physical therapy during the previous 3 months; (5) suffered an accident during the previous 6 months; (6) being pregnant; (7) being affected by a language restriction and comprehension; and (8) suffering neural alterations or cardiovascular diseases [18].

### 2.2. Instruments

#### 2.2.1. Musculoskeletal Discomfort

The Cornell Musculoskeletal Discomfort Questionnaire (CMDQ) was selected to assess changes in musculoskeletal discomfort. This is a valid and reliable tool in ergonomic risk evaluation [19]. This questionnaire evaluates, for a period of 7 days, musculoskeletal discomfort on an image of a body divided into 20 parts [20]. It is a researching rather than a diagnostic tool.

The CMDQ assesses 3 dimensions of musculoskeletal discomfort: (1) frequency; (2) discomfort; and (3) interference. Each dimension is evaluated using an ordinal scale. The frequency dimension is divided into 5 levels: never (0 points), 1–2 times per week (1.5 points), 3–4 times per week (3.5 points), every day (5 points), several times per day (10 points). The severity and interference dimensions are each divided into 3 levels. The result of the questionnaire is the sum of the points of the 3 dimensions.

The CMDQ is a weekly questionnaire, thus, to evaluate discomfort each day, the frequency dimension was held constant, and severity and interference were recalculated during the second evaluation. In our study, the neck and upper and lower back were evaluated.

#### 2.2.2. Muscle Tone

The muscle tone of the trapezius muscle was measured using a Myoton-Pro device (Myoton As, Tallinn, Estonia). This is a reliable, accurate, and noninvasive tool for measuring superficial muscle tone [21]. The device registers muscle oscillations to calculate its tone. Frequency is measured in hertz. The higher the frequency, the greater the muscle tone.

Five different points were assessed: (1) upper trapezius at the lateral third of the line between the acromion and C-7 spinous process; (2) upper trapezius at the line between the acromion and C-7 spinous process, at the superior edge of the scapula; (3) middle trapezius at T-5, between the spinous process and the scapula edge; (4) lower trapezius at T-8; and (5) lower trapezius at T-8, diagonally towards the lower edge of the scapula. Upper and lower trapezius tones are expressed as the mean of the values of all of the points assessed for each muscle [22,23]. Indirectly, asymmetry was considered as the tone difference between the right and left side in each muscular bundle.

#### 2.2.3. Procedure

Three evaluations were planned in this study: at the beginning (Pre_1) and at the end of the workday (Post_1), and 6 weeks after the training program (Pre_2), at the beginning of the workday. Musculoskeletal discomfort and muscle tone were measured at these times.

The training program was designed and supervised by a physiotherapist. He individually taught each worker the exercises and progressions, addressed their doubts, and registered how many days per week these exercises were carried out. The intervention lasted 6 weeks (2 training day/week). The training sessions were conducted at the workplace of each person for 5–15 min.

The exercises programmed for the intervention were based on 4 principal components [24,25,26]: (1) stretching of the cervical and/or dorsal region to decrease muscle rigidity; (2) joint mobility of shoulders and rachis; (3) strengthening of deep stabilizer and core muscles; and (4) scapula stabilizing exercises. The number of repetitions and load was individually adapted weekly to each worker. It was recommended to remain calm, control the natural curve of the spine, and to stop exercising if pain appeared.

#### 2.2.4. Data Analysis

Data are expressed as the mean and standard deviation (SD). The Kolmogorov–Smirnov test was applied to assess normality of the data. To evaluate changes in the results of the CMDQ, muscle tone, and symmetry between sides (right and left trapezius tone) at Pre_1, Post_1, and Pre_2, the paired Student’s t-test was applied to normally distributed variables and Wilcoxon’s W-test to the remainder. The significance level was set at *p* < 0.05. Additionally, the clinical changes were analyzed using Cohen’s d effect size (ES) in variables whose differences were statistically significant. The scale used to interpret Cohen’s d was d = 0.2–0.5 for a “small” ES, d = 0.5–0.8 for a “medium” ES, and d ≥ 0.8 for a “large” ES [27].

## 3. Results

Twenty-six participants that met the inclusion and exclusion criteria were recruited for the study.

The basal characteristics of the subjects are shown in Table 1. All participants were right-handed. The body mass index (BMI) showed a slight overweight condition (BMI > 25 kg/m^2^).

The results obtained in the previous workday evaluation (Pre_1) and after finishing the workday evaluation (Post_1) in the initial assessment are shown in Table 2.

The results do not show significant differences in musculoskeletal discomfort measured by the CMDQ for the back between the before and after workday evaluations. The values referring to the neck show a non-significant trend (*p* = 0.059) of increase in musculoskeletal discomfort during the day. In addition, no significant differences were found in muscle tone during the workday.

Nineteen participants followed the six week training program (7 males, 12 female). There were no differences in basal characteristics between the initial group (*n* = 26) and the group that followed the intervention proposed (*n* = 19).

Because there were no differences between pre-workday and post-workday evaluations in the different variables studied in the initial evaluation, in the post intervention assessment only the previous work evaluation was considered.

The results for the previous workday evaluation before intervention (Pre_1) and the previous workday evaluation after the six-week intervention (Pre_2) are shown in Table 3. The differences for all variables between the post-intervention and pre-intervention evaluations (Pre_2-Pre_1) are also included in the table (Table 3).

After the intervention program, the body mass index (BMI) significantly decreased (*p* < 0.03) with a trivial effect size (ES = −0.07). Moreover, the musculoskeletal discomfort measured with the CMDQ questionnaire significantly decreased in the neck (*p* = 0.027) and upper back (*p* = 0.046). The mean reduction of the discomfort was 1.8 for the neck and 2.05 for the upper back. The effect size observed was medium in both the neck (ES = −0.60) and upper back (ES = −0.67).

A significant decrease in the tone of the upper right trapezius (*p* = 0.003) with a medium effect size (ES = −0.66) was found, whereas the tone in the middle left trapezius was increased after the intervention (*p* = 0.016, ES = 0.45). The other sections of the trapezius studied did not show a significant change due to the intervention.

Before the intervention, the asymmetry (AS) was significant in the lower trapezius (AS = 0.77 ± 1.06; *p* = 0.005; ES = 0.22) and presented a non-significant trend in the upper trapezius (AS = 0.65 ± 1.44; *p* = 0.063; ES = 0.36). Both were considered small effect sizes. The middle trapezius did not show any significant asymmetry nor a considerable effect size (AS = −0.19 ± 1.55; *p* = 0.602; ES = −0.06).

This asymmetry disappeared after the intervention. The upper trapezius (AS = −0.35 ± 0.87; *p* = 0.095; ES = −0.19), middle trapezius (AS = −0.59 ± 1.84; *p* = 0.176; ES = −0.17), and lower trapezius (AS = 0.57 ± 2.25; *p* = 0.288; ES = 0.15) showed non-significant differences between the right and left sides and trivial effect sizes after completing the training program.

## 4. Discussion

The main findings of this study are that no significant increase in musculoskeletal discomfort or muscle tone of Trapezius were found during the workday (Table 2), and after a training period of 6 weeks, BMI, CMDQ results, and the upper trapezius tone were significantly lower (Table 3).

The increase in musculoskeletal discomfort during the workday in office workers can be influenced by numerous factors. Studies have found that placing the computer screen not in line with the eyes can be related to the appearance of neck pain or musculoskeletal discomfort [28,29]. From a functional perspective, Kocur et al. noted that clerks with pain in the cervical spine had advanced the position of the head in a sitting position and presented a higher rigidity in the upper trapezius, although there was no change in the pain threshold when pressing this muscles group [30]. In a more recent study, Kokur et al. assessed the relationship between advanced head posture, biomechanical parameters, and the pain threshold related to the pressure in superficial neck musculature in asymptomatic sedentary workers. Two groups of workers were distinguished (with and without forward head position) by measuring the tone at a point. The results did not find significant differences between groups for the upper trapezius. This appears to show that the forward head posture has no impact on muscular stiffness, or the tone and elasticity of the upper trapezius in healthy office workers [31]. In our study, the reasons for musculoskeletal discomfort in the neck and back were not analyzed. The working desk used was ergonomically designed, thus, the prolonged use of the computer may be the cause of this discomfort as suggested by other studies [32].

In reference to the muscular tone, a decrease in the upper trapezius of the dominant arm and a smaller increase in the left trapezius were found in this study (Table 3). The asymmetries between right and left sides decreased after the intervention program. These asymmetries in tone could be due to the use of the mouse [33].

Several investigations have shown how different exercises affect functionality and the quality of life of office workers [34,35]. Various studies have concluded that neck and shoulder stretching exercises can improve the functionality of the neck and the quality of life of the office workers [8,36]. Nonetheless, other studies note that beneficial health effects cannot by obtained solely with stretching exercises. In addition, Petersen et al. showed that people with neck pain often have debility in the scapulothoracic muscles [37]. Studies in which stretching combined with active strengthening exercise is proposed, such as ours, have shown better results [26,38]. Andersen et al. investigated if 10 weeks of training of the scapular function (including upper trapezius) was effective in decreasing pain in the neck and shoulder regions in adults [39]. In our research, the exercises included followed the objective of training the scapular function and reducing the upper trapezius activation. This program may have contributed to reducing musculoskeletal discomfort in the neck, increasing the tone of the middle trapezius, and reducing the muscle tone in the upper trapezius. In contrast to these authors, no changes were found in the lower trapezius tone in our study. 

In addition to all of the previous factors noted above, Ebaugth et al. showed that fatigue of the external rotator muscles of the shoulder can alter the kinematics of the shoulder gridle [40]. This was considered in our study, thus, exercises for strengthening of the shoulder rotator cuff were included. The objective of this intervention is to provoke its activation and to increase its endurance, thus avoiding early fatigue. This should also increase the shoulder’s stability.

Recently, Thongprasert et al. highlighted an important association in the muscle control between cervical and lumbar stabilizers, showing an abnormal performance in cervical stabilizer muscles in people with lumbar pain [41]. The program of exercise included both cervical stabilizers and exercises aimed to improve central stability with core activation, considering this relationship to stimulate strengthening and higher stability. Finally, Shariat et al. proposed an exercise program designed to reduce the muscular stiffness and pain in the workplace [17].

Considering the previous data, an exercise program based on four main components (scapular stability, cervical and dorsal stretching, mobility exercise of the back and shoulders, and strengthening of the cervical, shoulder and core stabilizer muscles) can reduce musculoskeletal discomfort and adjust the trapezius muscle tone [24,25,26,38], as shown in our data.

Although several studies exist that describe exercise programs for reducing neck pain and musculoskeletal discomfort, their effectiveness has not been verified. The optimal frequency and duration of the exercises, in addition to the importance of their supervision, are not known [18]. In most previous studies, the intervention time ranges from 4 weeks to a year with a frequency between 2 and 3 times per week [42]. The obtained results in this research, using a six week supervised program and frequency of 2 days per week, appears to be consistent with programs analyzed in the scientific literature.

In addition, studies conducted with interventions not based on therapeutic exercise have also been shown to be effective in reducing musculoskeletal discomfort. Ting et al. showed that an intervention with a training program did not obtain significantly better effects than a combined intervention of ergonomic and health promotion [43]. Therefore, it is considered necessary to continue developing MSD prevention programs in the work environment by integrating all tools that can be effective in this context.

## 5. Conclusions

In conclusion, musculoskeletal discomfort in office workers is constant during the workday, but can be improved if a 6 week specific training program is applied. The trapezius muscle tone did not change during the workday, although the right upper trapezius tone decreased and the left middle trapezius tone increased after a 6 week training program, which reduced muscle asymmetries and may explain the reduction of musculoskeletal discomfort.

There are some limitations in this study. The first is the lack of a control group and the small sample size. Due to company demands, it was more suitable to develop a training program with all of the volunteers that participated in the study. A larger sample size could have helped to obtain more reliable conclusions. The second limitation is the absence of a follow-up of the training. Adherence and autonomy of the workers after the program, without supervision, could have been analyzed.

Due to the small number of studies about the prevention of musculoskeletal discomfort in office workers, this research could be considered a guide for future interventions with similar objectives. Workplace exercise intervention programs that include stretching of cervical and dorsal regions, mobility of shoulders and rachis, strengthening of deep stabilizers and core muscles, and scapular stabilization could be suitable for the reduction of neck and back discomfort. The reduction of discomfort in workers could translate into a reduction of sick leave due to musculoskeletal disorders and an increase in worker productivity. The exercise routine should be supervised by a sport science professional or physiotherapist to control the exercise execution.

This low-cost intervention, performed in the workplace and during work time, which can be developed in small groups, could also stimulate other psychosocial variables.

## Figures and Tables

**Table 1:** Basal characteristics of the participants (*n* = 26). Data are expressed as mean ± SD.

Age (years)	40.04 ± 10.6
Years working	15 ± 10.8
Mouse laterality	100% right
Height (meters)	1.7 ± 0.1
Weight (kg)	69.6 ± 11.8
BMI (kg/m^2^)	25.2 ± 3.6
BMI: body mass index	

**Table 2:** Musculoskeletal discomfort and muscle tone in the initial assessment in pre and post workday evaluations (*n* = 26). Data are expressed as mean ± SD.

	Pre_1	Post_1	Post_1-Pre_1	*p*
CMDQ				
CMDQ Neck	2.45 ± 3.96	2.84 ± 1.09	0.39 ± 1.34	0.059
CMDQ Upper Back	2.04 ± 3.72	1.75 ± 1.41	−0.29 ± 1.59	0.461
CMDQ Lower Back	0.7 ± 2.23	1.15 ± 2.54	0.45 ± 2.58	0.593
Muscular Tone (Hz)				
Upper RIGHT Trapezius	15.43 ± 1.77	15.48 ± 1.77	0.05 ± 0.84	0.765
Middle RIGHT Trapezius	18.24 ± 2.87	18.51 ± 2.83	0.27 ± 1.06	0.218
Lower RIGHT Trapezius	20.24 ± 3.47	20.43 ± 3.28	0.19 ± 1.17	0.425
Upper LEFT Trapezius	15.02 ± 1.71	14.94 ± 2.06	−0.08 ± 1.79	0.818
Middle LEFT Trapezius	18.52 ± 2.87	18.45 ± 2.88	−0.07 ± 1.67	0.828
Lower LEFT Trapezius	19.94 ± 3.03	20.12 ± 2.85	0.17 ± 1.21	0.482

Pre_1: Pre-workday initial assessment; Post_1: post-workday initial assessment; CMDQ: Cornell Musculoskeletal Discomfort Questionnaire.

**Table 3:** Musculoskeletal discomfort and muscular tone in pre- and post-workday assessment after the intervention evaluation (*n* = 19). Data are expressed as mean ± SD.

	Pre_1	Pre_2	Pre_2-Pre_1	*p*	ES
BMI (kg/m^2^)	25.37 ± 3.60	25.12 ± 3.51	−0.25 ± 0.44	0.030 *	−0.07
CMDQ					
CMDQ Neck	2.58 ± 3.93	0.77 ± 1.61	−1.8 ± 3.16	0.027 *	−0.60
CMDQ Upper Back	2.55 ± 4.18	0.5 ± 1.04	−2.05 ± 4.05	0.046 *	−0.67
CMDQ Lower Back	0.69 ± 2.39	0.08 ± 0.35	−0.61 ± 2.05	0.180	−0.36
Muscular Tone (Hz)					
Upper RIGHT Trapezius	15.61 ± 1.89	14.42 ± 1.98	−1.29 ± 1.58	0.003 *	−0.66
Middle RIGHT Trapezius	18.33 ± 3.06	19.36 ± 3.38	+1.08 ± 2.36	0.068	0.33
Lower RIGHT Trapezius	20.31 ± 3.44	20.38 ± 3.43	+0.14 ± 1.92	0.750	0.10
Upper LEFT Trapezius	14.96 ± 1.77	14.78 ± 1.64	−0.23 ± 1.39	0.478	−0.14
Middle LEFT Trapezius	18.52 ± 3.01	19.95 ± 3.57	+1.52 ± 2.42	0.016 *	0.45
Lower LEFT Trapezius	19.54 ± 3.12	19.81 ± 4.20	+0.33 ± 2.61	0.589	0.09

Pre_1: pre-workday initial assessment; Pre_2: pre-workday after intervention assessment; BMI: body mass index; CMDQ: Cornell Musculoskeletal Discomfort Questionnaire; ES: Effect Size * *p* < 0.05.
